# Genetic Variants Associated with Non-Steroidal Anti-Inflammatory Drug-Induced Stevens–Johnson Syndrome and Toxic Epidermal Necrolysis

**DOI:** 10.3390/medsci14010098

**Published:** 2026-02-19

**Authors:** Jenita Kosanlawit, Parinya Konyoung, Warayuwadee Amornpinyo, Wichittra Tassaneeyakul, Sirimas Kanjanawart, Oranuch Pattanacheewapull, Danklai Purimart, Nontaya Nakkam

**Affiliations:** 1 Department of Pharmacology, Faculty of Medicine, Khon Kaen University, Khon Kaen 40002, Thailand; jenita.jj@kkumail.com (J.K.); wichittra.tassaneeyakul@gmail.com (W.T.); sirimas@kku.ac.th (S.K.); 2 Pharmacy Unit, Udon Thani Hospital, Udon Thani 41000, Thailand; panyatree1@gmail.com (P.K.); filmphy03@gmail.com (D.P.); 3 Division of Dermatology, Department of Internal Medicine, Khon Kaen Hospital, Khon Kaen 40000, Thailand; warayuwadee.a@gmail.com; 4 Department of Pharmacy, Khon Kaen Hospital, Khon Kaen 40000, Thailand; oranuit996@gmail.com

**Keywords:** non-steroidal anti-inflammatory drugs, piroxicam, Stevens–Johnson syndrome, toxic epidermal necrolysis, pharmacogenetics, human leukocyte antigen (*HLA*), Cytochrome P450 2C9 (*CYP2C9*)

## Abstract

**Background/Objectives**: Non-steroidal anti-inflammatory drugs (NSAIDs) are widely prescribed to help alleviate pain and treat inflammation, but they are also recognized as common causes of severe cutaneous adverse reactions (SCARs), including Stevens–Johnson syndrome (SJS) and toxic epidermal necrolysis (TEN). Despite their clinical importance, pharmacogenetic markers to predict individual susceptibility to NSAID-induced SJS/TEN remain insufficiently defined. This study investigated associations between *HLA* class I and II alleles, *CYP2C9* polymorphisms, and NSAID-induced SJS/TEN in a Thai population. **Methods**: A total of 18 patients with NSAID-induced SJS/TEN and 54 NSAID-tolerant controls were enrolled. Genotype data from 183 unrelated Thai individuals without a history of drug allergy were included as a general population control group. Genotyping was performed for *HLA* class I and II alleles and the CYP2C9*3 variant. **Results**: HLA-DQB1*03:02 was significantly associated with NSAID-induced SJS/TEN (OR = 9.23, 95% CI = 2.19–38.83, *p* = 0.0024, Pc = 0.0312), particularly those triggered by piroxicam (OR = 13.71, 95% CI = 2.81–66.86, *p* = 0.0012, Pc = 0.0156). Additional associations were identified for HLA-B*56:01 and HLA-A*68:01 in the overall NSAID-induced SJS/TEN group. The subgroup analysis suggested that these alleles, along with HLA-DRB1*04:03, were associated with an increased risk of piroxicam-induced SJS/TEN. However, these associations did not remain statistically significant after Bonferroni’s correction. No significant association was identified for CYP2C9*3. **Conclusions**: This study identified specific *HLA* alleles, particularly HLA-DQB1*03:02, as candidate pharmacogenetic risk factors for NSAID-induced SJS/TEN in a Thai population, especially in piroxicam-associated cases. However, these associations should be considered exploratory. Larger, multicenter, multi-ethnic studies are required to validate these findings and clarify their potential clinical utility.

## 1. Introduction

Non-steroidal anti-inflammatory drugs (NSAIDs) are widely prescribed for the treatment of inflammation and pain; consequently, NSAID-induced adverse drug reactions (ADRs) are frequently reported across populations [1,2]. These ADRs include predictable, dose-dependent type A reactions and unpredictable, immune-mediated type B reactions [3]. Among type B reactions, cutaneous adverse drug reactions are the most frequent, which represent various conditions ranging in severity from mild, self-limited cutaneous eruptions such as maculopapular exanthema (MPE) to severe cutaneous adverse reactions (SCARs), particularly Stevens–Johnson syndrome (SJS) and toxic epidermal necrolysis (TEN). These reactions represent life-threatening delayed type IV hypersensitivity reactions mediated by T cells, typically developing days to weeks after drug exposure [4]. Although the overall incidence of SCARs is low, the SJS/TEN phenotype is associated with the highest mortality. SJS carries a mortality rate of approximately 5–20%, while that of TEN can reach up to 50% [4,5]. Moreover, patients who recover from SJS/TEN episodes may be left with sequelae or long-lasting disabilities such as visual impairment or skin sensitivity [4,5,6].

A previous study of 1028 Asian patients with SJS/TEN, including Thai individuals, reported that NSAIDs were among the most common causative drug groups, accounting for approximately 4% of cases, following antiepileptic drugs/antipsychotics (53%), antibiotics/antiviral agents (20%), and allopurinol (19%) [7]. A recent study performed in Thailand, which included a total of 480 patients with probable or definite SCARs, reported an overall incidence of NSAID-induced SCARs of approximately 6.9%, with SJS/TEN accounting for about 8.3% of all drug-induced SJS/TEN cases [8]. Specific NSAIDs such as piroxicam, diclofenac, and COX-II inhibitors, including etoricoxib and celecoxib, showed a high incidence of drug-induced SJS/TEN [7]. Moreover, a previous study performed in Korea reported the phenotypes of SCARs induced by NSAIDs and their causative drugs; the results showed that propionic acid derivatives were the predominant NSAID class in SJS/TEN [9]. Despite the clinical importance of NSAIDs as triggers of SJS/TEN, the underlying genetic susceptibility factors remain poorly defined compared with those of other drug classes.

Human leukocyte antigen (*HLA*) variation is a key risk determinant for drug-induced SCARs [3,4,5]. Well-established examples include HLA-B*15:02 for carbamazepine [10,11], HLA-B*58:01 for allopurinol [12], or HLA-B*57:01 for abacavir hypersensitivity [13,14]. These discoveries highlight the strong immunogenetic basis of SCARs and the clinical utility of *HLA*-based pharmacogenetic screening. However, in contrast to these well-characterized associations, evidence linking *HLA* alleles to NSAID-induced SJS/TEN remains limited and inconsistent. A European study reported an association between HLA-B*73:01 and oxicam NSAID-induced SJS/TEN [15], while other studies of cold medicine-related SJS/TEN with severe ocular surface complications (SOCs) identified associations with HLA-B*44:03 and related haplotypes in several populations including Japanese, Indian, Brazilian [16,17], and Thai individuals [18], in which cold medicines refer to NSAIDs and multi-ingredient cold medications (antipyretics or analgesics). These phenotypes, however, often involve multi-ingredient formulations and are not specific to NSAID-induced SJS/TEN. Thus, the genetic factors predisposing individuals to true NSAID-induced SJS/TEN remain largely unknown.

In addition to immunogenetic predisposition, variability in drug metabolism may contribute to susceptibility to SCARs. Cytochrome P450 2C9 (CYP2C9) is the primary enzyme responsible for metabolizing several NSAIDs such as piroxicam, which is metabolized primarily via 5′-hydroxylation by CYP2C9, accounting for approximately 60% of its metabolic clearance [19,20]. Reduced-function *CYP2C9* alleles, particularly CYP2C9*3, markedly decrease enzymatic activity and have been associated with an increased risk of phenytoin-induced SCARs in Asian populations [21,22,23]. Impaired metabolism could lead to higher systemic NSAID exposure, thereby increasing the risk of adverse reactions, including immune-mediated toxicity [24,25]. Although the Clinical Pharmacogenetics Implementation Consortium (CPIC) provides therapeutic recommendations for NSAIDs based on *CYP2C9* genotypes to optimize efficacy and minimize toxicity [26], evidence regarding the association of *CYP2C9* variants with NSAID-induced SJS/TEN remains limited.

Given the serious implications of NSAID-induced SJS/TEN and the emerging yet inconclusive evidence on genetic susceptibility, this study aimed to investigate the associations between genetic polymorphisms in *HLA* class I and II genes and NSAID-metabolizing enzyme genes with NSAID-induced SJS/TEN in a Thai population.

## 2. Materials and Methods

### 2.1. Study Population

This study employed a prospective, hospital-based case–control design to investigate the genetic risk factors associated with NSAID-induced SJS/TEN. Patients clinically diagnosed with SJS, SJS/TEN overlap, or TEN attributed to NSAID exposure and admitted to local hospitals in Thailand between 2008 and 2024 were prospectively enrolled. The initial diagnosis of SJS/TEN was made by an internist or dermatologist at each hospital and subsequently confirmed by a dermatologist from the investigator team. Diagnosis was based on clinical features, including the pattern of skin eruptions, percentage of skin detachment, timing between drug exposure and onset of SJS/TEN, and laboratory results. Clinical manifestations of SJS/TEN typically include prodromal symptoms (e.g., fever, sore throat, and early mucosal involvement), atypical target lesions, positive Nikolsky’s sign, epidermal detachment, and mucosal erosions, particularly affecting the eyes, mouth, and genitalia [4]. To ensure the accurate identification of causative drugs, NSAID causality was determined using both the Naranjo adverse drug reaction (score ≥ 5, probable ADR) [27] and the algorithm for the assessment of drug causality (ALDEN score ≥ 4; probable SJS/TEN) [28]. Eligible cases required a well-documented history of NSAID exposure within a compatible latency period, complete clinical information for causality assessment, and no alternative diagnoses or confounding dermatologic or autoimmune conditions. NSAID-tolerant controls were recruited from the same hospitals and had used NSAIDs on multiple occasions for a cumulative duration of over 180 days without any hypersensitivity reactions. Individuals with incomplete records, underlying dermatologic disease, or a history of hypersensitivity to other medications were excluded. In addition to NSAID-tolerant controls, the genotyped data obtained from 183 unrelated native Thai individuals without any evidence of drug hypersensitivity, as previously published [29], were included as the general Thai population control.

All participants were informed verbally and in writing about the study procedures and objectives, and written informed consent was obtained. The study protocol was approved by the Ethics Committee for Human Research, Khon Kaen University, Thailand (HE510837).

### 2.2. Genomic DNA Preparation

Peripheral blood samples were collected in EDTA-coated tubes. Leukocytes were separated via centrifugation at 3500 rpm for 15 min, and genomic DNA was subsequently extracted using QIAamp^®^ DNA blood mini kits (QIAGEN^®^ GmbH, Hilden, Germany).

### 2.3. HLA Genotyping

Genotyping of *HLA* class I, including *HLA-A*, *HLA-B*, and *HLA-C*, and *HLA* class II, including *HLA-DRB1*, *HLA-DQA1*, and *HLA-DQB1*, was determined using the LIFECODES^®^ *HLA* Typing Kits (Immucor GTI Diagnostics, Waukesha, WI, USA), where a polymerase chain reaction–sequence-specific oligonucleotide probe method (PCR-SSOP) and xMAP technology, designed to be used with the Luminex^®^ system, were utilized, as previously described [30]. *HLA* alleles were assigned using LIFECODES^®^ MATCH IT DNA Software version 1.3 based on Allele Database version 3.50. The genotyping results were reported at intermediate-to-high resolution (four-digit or two-field level).

### 2.4. CYP2C9 Genotyping

CYP2C9*3 (rs1057910) was genotyped using TaqMan^™^ Drug Metabolism Genotyping Assays (assay ID: C__27104892_10, Applied Biosystems, Foster City, CA, USA on a QuantStudio^™^ 6 Flex Machine).

### 2.5. Data and Statistical Analysis

The demographic data and clinical characteristics of NSAID-induced SJS/TEN cases and the control groups were assessed using descriptive statistics, including percentages, medians with ranges, and means with standard deviations. Comparisons of clinical characteristics between cases and controls were performed using Student’s two-tailed *t*-tests for continuous variables and Fisher’s exact tests for categorical variables.

To study the association between *HLA* and *CYP2C9* genetic polymorphisms and NSAID-induced SJS/TEN, the allele frequencies and carrier frequencies of the *HLA* class I and II alleles and the CYP2C9*3 variant were determined by direct counting and compared with the control groups using Fisher’s exact test. The strength of the associations was estimated by odds ratios (ORs) and 95% confidence intervals (CIs) using SPSS statistical software, version 28.0 for macOS (IBM, Armonk, NY, USA). ORs were determined using Haldane’s modification, which added 0.5 to all cells to accommodate possible zero counts [31]. To address multiple comparisons, Bonferroni’s correction (corrected *p*-values, Pc) was applied based on the number of alleles tested within each *HLA* locus (21 for *HLA-A*, 35 for *HLA-B*, 19 for *HLA-C*, 22 for *HLA-DRB1*, 9 for *HLA-DQA1*, 13 for *HLA-DQB1*, and 2 for CYP2C9*3). All *p*-values were empirical, two-tailed values, and a value of less than 0.05 was considered statistically significant.

## 3. Results

### 3.1. Characteristics of the Study Population

Of the NSAID-induced SJS/TEN cases identified, 18 patients met the inclusion criteria and were enrolled in the study. Among these 18 patients, 14 patients (77.78%) were diagnosed with SJS, 1 patient (5.55%) with SJS/TEN overlap, and 3 patients (16.67%) with TEN. NSAID-induced SJS/TEN cases included 9 males (50%) and 9 females (50%), with a mean age of 51.61 ± 18.38 years. The mean exposure time of the NSAIDs until symptom onset (latency period) of SJS/TEN or onset of SJS/TEN was 7.06 ± 9.41 days (ranging from 1 to 30 days) after drug initiation. The data are presented in Table 1. As a comparison group, 54 NSAID-tolerant individuals were enrolled as controls; they received NSAIDs on multiple occasions for a cumulative duration exceeding 180 days without any hypersensitivity reactions. This group comprised 10 males (18.52%) and 44 females (81.48%), with a mean age of 60.43 ± 10.12 years, as shown in Table 1.

Laboratory findings and mucosal involvement are summarized in Table 1. Among patients with NSAID-induced SJS/TEN, elevated liver transaminase levels (AST and ALT ≥ 3-fold above the upper limit of normal) were observed in two SJS cases. Renal insufficiency, defined as serum creatinine > 2.25 mg/dL at onset, was identified in one patient with SJS/TEN overlap, and eosinophilia (≥ 5%) was detected in two patients with SJS (14.29%). Mucosal involvement was frequently observed, including in the oral (15/18, 83.33%), ocular (11/18, 61.11%), and genital (5/18, 27.78%) regions.

The average duration of hospital stay for the treatment of SJS/TEN was 19 ± 29.56 days (ranging from 3 to 86 days), with the longest average duration observed in patients with the SJS/TEN overlap phenotype. The mean hospitalization cost was 40,852.79 ± 117,854.09 Thai baht (ranging from 223 to 449,342 Thai baht). Among the 18 NSAID-induced SJS/TEN cases, no in-hospital mortality was reported (Table 1).

In this study, piroxicam was identified as the leading causative NSAID, accounting for the majority of SJS/TEN cases (11/18 cases, 61.11%), followed by celecoxib (2 cases), ibuprofen (2 cases), diclofenac, metamizole, and naproxen, as shown in Table 2.

### 3.2. Associations Between HLA Alleles and NSAID-Induced SJS/TEN

*HLA* class I and II genotyping was performed for each individual in this study. A total of 21 *HLA-A* alleles, 35 *HLA-B* alleles, 19 *HLA-C* alleles, 22 *HLA-DRB1* alleles, 9 *HLA-DQA1* alleles, and 13 *HLA-DQB1* alleles were identified among the study population, which included 72 participants comprising both cases and tolerant controls. The distribution of *HLA* class I and II alleles observed in patients with NSAID-induced SJS/TEN and NSAID-tolerant controls was summarized and is outlined in Supplementary Tables S1 and S2. Among *HLA* class I loci, the most common alleles based on carrier frequency in NSAID-induced SJS/TEN cases were HLA-A*11:01 (55.56%), HLA-B*15:02 (22.22%), and HLA-C*01:02 (33.33%), whereas in NSAID-tolerant controls, the most frequent carriers were HLA-A*11:01 (42.59%), HLA-B*46:01 (25.93%), and HLA-C*07:02 (33.33%). For *HLA* class II loci, the most common alleles based on carrier frequency in NSAID-induced SJS/TEN cases were HLA-DRB1*15:02 (27.78%), HLA-DQA1*01:01 (38.89%), and HLA-DQB1*03:01, HLA-DQB1*03:02, and HLA-DQB1*05:01 (27.78%), whereas in NSAID-tolerant controls, the most frequent carriers were HLA-DRB1*04:05 (48.15%), HLA-DQA1*01:01 (46.30%), and HLA-DQB1*05:01 (42.59%). No previously unreported *HLA* variants were detected based on the genotyping resolution applied in this study.

Comparison of *HLA* genotyping data between patients with NSAID-induced SJS/TEN and control groups revealed that the carrier frequencies of three *HLA* alleles, including HLA-DQB1*03:02, HLA-A*68:01, and HLA-B*56:01, were higher in the NSAID-induced SJS/TEN group (*p* < 0.05; Table 3). Among these three alleles, HLA-DQB1*03:02 was statistically significantly associated with an increased risk of NSAID-induced SJS/TEN when compared with the general Thai population, with an OR of 9.23 (95% CI = 2.19–38.83, *p* = 0.0024, Pc = 0.0312). The frequency of HLA-DQB1*03:02 carriers in the NSAID-induced SJS/TEN group was 27.78%, approximately 6.94 times higher than that observed in the general Thai population (4%), as shown in Table 3. Compared with NSAID-tolerant controls, carriers of HLA-DQB1*03:02 demonstrated a higher carrier frequency among SJS/TEN cases (27.78% vs. 14.81%), corresponding to an increased risk (OR = 2.21, 95% CI = 0.62–7.92, *p* = 0.2886). In addition to HLA-DQB1*03:02, the frequency of HLA-B*56:01 carriers among patients with NSAID-induced SJS/TEN was approximately 10.10-fold higher (11.11%) than that in the general Thai population (1.10%). Similarly, the frequency of HLA-A*68:01 carriers was approximately 8.17-fold higher in the NSAID-SJS/TEN group (16.67%) compared with that in the general population (2.04%). Correspondingly, increased risks of NSAID-induced SJS/TEN were observed in individuals carrying HLA-B*56:01 (OR = 11.31, 95% CI = 1.49–85.75, *p* = 0.0189, Pc = 0.6615) and HLA-A*68:01 (OR = 8.95, 95% CI = 1.83–43.75, *p* = 0.0068, Pc = 0.1428). Comparisons with NSAID-tolerant controls demonstrated similar patterns of increased carrier frequencies and risks of carrying these additional alleles; however, these associations did not reach statistical significance, so they should be interpreted as exploratory (Table 3). Taken together, these results highlight HLA-DQB1*03:02 as the strongest and most consistent risk signal for NSAID-induced SJS/TEN in this study, while HLA-B*56:01 and HLA-A*68:01 represent additional exploratory associations.

Subgroup analysis focusing on piroxicam, the major causative NSAID in this study, revealed that the frequency of HLA-DQB1*03:02 carriers was approximately 9.09-fold higher among patients with piroxicam-induced SJS/TEN (36.36%) compared with the general Thai population (4%). This allele was statistically significantly associated with an increased risk of piroxicam-induced SJS/TEN, with an OR of 13.71 (95% CI = 2.81–66.86, *p* = 0.0012, Pc = 0.0156). When compared with NSAID-tolerant controls, a similar trend toward increased carrier frequency and risk was observed among HLA-DQB1*03:02 carriers (OR = 3.29, 95% CI = 0.78–13.86, *p* = 0.1054, Pc = 1.3702), as presented in Table 4. Furthermore, the frequencies of HLA-B*56:01 and HLA-A*68:01 carriers among patients with piroxicam-induced SJS/TEN were approximately 16.53-fold (18.18%) and 8.91-fold (18.18%) higher, respectively, compared with the general Thai population (1.10% and 2.04%). These alleles were associated with increased risks of SJS/TEN induced by piroxicam (HLA-B*56:01: OR = 20.11, 95% CI: 2.53–159.56, *p* = 0.0045, Pc = 0.1575; HLA-A*68:01: OR = 9.94, 95% CI: 1.60–61.66, *p* = 0.0136, Pc = 0.2856); however, neither association remained statistically significant after Bonferroni’s correction. Comparisons with NSAID-tolerant controls demonstrated similar patterns of increased carrier frequencies and risks of these additional alleles; however, these associations did not reach statistical significance (Table 4). Interestingly, carriers of HLA-DRB1*04:03 also demonstrated a higher risk of piroxicam-induced SJS/TEN compared with both tolerant controls and the general Thai population controls, with ORs ranging from 13.33 to 16.50, as shown in Table 4. Collectively, these findings highlight HLA-DQB1*03:02 as the most prominent genetic risk signal for piroxicam-induced SJS/TEN, while HLA-DRB1*04:03, HLA-B*56:01, and HLA-A*68:01 appear to confer additional exploratory risk associations.

### 3.3. Associations Between CYP2C9 Polymorphisms and NSAID-Induced SJS/TEN

CYP2C9*3 genotypes were analyzed in all patients. The frequencies of CYP2C9*1/*1, CYP2C9*1/*3, and CYP2C9*3/*3 among patients with NSAID-induced SJS/TEN were not significantly different from those observed in tolerant controls or the general Thai population. No significant association was observed between CYP2C9*3 polymorphism and the risk of NSAID-induced SJS/TEN. Subgroup analysis of patients exposed to piroxicam demonstrated consistent results, with no significant difference in CYP2C9*3 genotype distribution compared with the controls, as summarized in Table 5.

## 4. Discussion

In this study, the associations between NSAID-induced SJS/TEN and genetic polymorphisms in *HLA* and NSAID-metabolizing enzyme genes were comprehensively investigated in a cohort comprising 18 SJS/TEN patients, 54 NSAID-tolerant controls, and 183 individuals from the general Thai population [29]. *HLA* class I (*HLA-A*, *-B*, and *-C*) and class II (*HLA-DRB1*, *-DQA1*, and *-DQB1*) loci were selected for analysis because previously reported pharmacogenetic risk alleles associated with drug-induced SCARs have been predominantly identified within these regions [5]. However, additional class II regions, including *HLA-DP*, were not evaluated and may represent areas for future investigation.

Consistent with previous studies in European [33,34] and Asian [7] populations that identified oxicam NSAIDs, particularly piroxicam, as common causative drugs of SJS/TEN, piroxicam was also the most frequent causative drug in this study. The clinical manifestations observed were typical of SJS/TEN, characterized by early mucosal involvement and extensive epidermal detachment, with a mean latency of approximately 7 days (ranging from 1 to 30 days) following NSAID exposure.

To the best of our knowledge, this is the first and largest well-defined case–control study to investigate the association of both *HLA* class I and II alleles, as well as *CYP2C9* polymorphisms, with NSAID-induced SJS/TEN. Among the genetic variants analyzed, HLA-DQB1*03:02 demonstrated the strongest suggestive association with NSAID-induced SJS/TEN. The direction and magnitude of this association were consistent when compared with both NSAID-tolerant controls, the most clinically relevant reference group, and the general Thai population, which reflects baseline allele frequencies, thereby supporting a potential underlying biological signal. However, statistical significance was achieved only when cases were compared with the general population and not with the tolerant control group. This discrepancy may be explained by the limited sample size of the tolerant control cohort; therefore, these findings should be interpreted as exploratory. Additional trends were observed for HLA-B*56:01 and HLA-A*68:01, which also appeared to be more frequent among affected individuals. These findings differ from those reported in a Taiwanese study [35], which identified HLA-A*02:01, HLA-A*34:01, HLA-B*46:01, and HLA-DPB1*02:02 as risk alleles for a broader spectrum of NSAID hypersensitivity, including immediate, delayed, and pseudo-allergic reactions, differences in phenotype that may account for the discrepant associations. The results from this study also differ from those generated in studies of cold medicine-related SJS/TEN with severe ocular complications, in which population-specific risk alleles have been reported, such as HLA-A*02:06 and HLA-B*44:03 in Japanese patients [16], HLA-A*02:07 and HLA-B*46:01 in Han Chinese patients [36], and HLA-B*44:03 and HLA-C*07:01 in Thai patients [18]. Overall, the differing genetic associations across studies underscore the importance of considering drug specificity, clinical phenotype, and ethnic background when evaluating genetic susceptibility to SCARs.

Subgroup analysis of piroxicam-induced SJS/TEN showed that carriers of HLA-DQB1*03:02 had a significantly increased risk of developing this reaction, which is consistent with the overall association observed for NSAID-induced SJS/TEN. Notably, the allele frequency of HLA-DQB1*03:02 is relatively high across various populations, estimated at approximately 4.26% in Thai individuals [32], 5.75% in Chinese individuals [37], and 9.62% in Korean individuals [38], supporting its possible clinical relevance. These observations suggest that HLA-DQB1*03:02 genotyping may have emerging relevance for identifying individuals at heightened risk of NSAID-induced SJS/TEN, particularly when piroxicam is being considered. In addition to HLA-DQB1*03:02, *HLA* class I alleles, including HLA-B*56:01 and HLA-A*68:01, were apparently associated with NSAID-induced SJS/TEN and piroxicam-induced SJS/TEN. However, these associations differ from those previously reported in European populations. For example, a study by Lonjou et al. [15] identified a strong association between HLA-B*73:01 and oxicam-induced SJS/TEN. Notably, HLA-B*73:01 was not detected in our Thai cohort or in previous studies with a Thai population [29,32]. In contrast, the allele frequencies of HLA-B*56:01 and HLA-A*68:01 in the Thai population were relatively low, at approximately 1–2% [32]. These findings underscore the critical importance of ethnicity-specific pharmacogenetic research, as genetic risk factors for NSAID-induced SJS/TEN, particularly those associated with piroxicam, may vary substantially across populations. Incorporating population-specific pharmacogenetic data into clinical decision-making may enhance precision medicine approaches and improve the safety of NSAIDs being prescribed across diverse ethnic groups.

Furthermore, carriers of the HLA-DRB1*04:03 allele exhibited an increased risk of developing piroxicam-induced SJS/TEN compared with both tolerant controls and the general Thai population. Interestingly, HLA-DRB1*04:03 has also been reported to be one of the genetic susceptibilities in patients with drug-induced liver injury attributed to NSAIDs (NSAID-DILI) in the United States [39]. This phenotype represents an idiosyncratic, likely immune-mediated form of liver injury that occurs in genetically predisposed individuals upon NSAID exposure [39,40,41]. Collectively, these findings support a broader role for HLA-DRB1*04:03 in conferring susceptibility to NSAID-induced immune-mediated adverse reactions.

Mechanistically, HLA class II molecules present exogenous or drug-modified peptides to CD4^+^ T lymphocytes, leading to cytokine production and the activation of immune pathways characteristic of delayed-type hypersensitivity reactions, particularly SCARs. In parallel, HLA class I molecules present drug-derived or haptenated peptides to CD8^+^ cytotoxic T cells, the primary effector cells responsible for keratinocyte apoptosis in SJS/TEN. Prior studies have demonstrated that coordinated CD4^+^ and CD8^+^ T-cell activation forms the immunological basis of several drug-induced SCAR phenotypes, including SJS/TEN and DRESS [10,12,13]. Supporting this concept, in vitro studies have shown cross-reactivity between HLA-A*32:01-restricted vancomycin-induced DRESS and other glycopeptide antibiotics, which was observed predominantly in patients who shared an *HLA* class II haplotype (HLA-DQA1*01:01 and HLA-DQB1*05:03) [42]. In the context of our findings, HLA-DQB1*03:02 demonstrated the strongest suggestive association with NSAID-induced SJS/TEN, particularly in piroxicam-related cases, whereas additional alleles, including HLA-B*56:01, HLA-A*68:01, and HLA-DRB1*04:03, showed more modest associations. Therefore, concurrent involvement of both *HLA* class II and class I alleles may reflect their performance of complementary roles in antigen presentation and T-cell activation. However, given the limited sample size and the loss of statistical significance after Bonferroni’s correction, these mechanistic interpretations should be considered exploratory, and functional studies are required to clarify the immunopathogenic relevance of these genetic associations.

Although individual *HLA* alleles were analyzed in this study, we acknowledge that *HLA* haplotypes may also contribute to the immunogenetic susceptibility to SJS/TEN. Certain combinations of *HLA* class I and class II alleles can influence antigen processing and T-cell activation in these reactions. In this study, we explored the haplotype frequencies of the identified risk alleles (e.g., HLA-A*68:01-HLA-B*56:01-HLA-DQB1*03:02); however, these multi-locus haplotypes occurred at very low frequencies in both cases and controls.

In addition to *HLA* variation, the potential contribution of drug-metabolizing enzyme polymorphisms to NSAID-induced SJS/TEN was considered in this study. Previous studies in Asian populations have shown that CYP2C9*3 markedly increased the risk of phenytoin-induced SCARs [21,22,23], and CYP2C9 plays an important role in the metabolism of several NSAIDs, particularly piroxicam. The CYP2C9*3 allele results from an A1075C substitution in exon 7, leading to an amino acid change from isoleucine to leucine at position 359, and it has been shown to reduce enzymatic clearance by approximately 90% compared with the wild-type allele [43]. Therefore, *CYP2C9* genetic polymorphisms may influence drug biotransformation and potentially affect both the efficacy and safety of NSAID therapy [26]. Although CYP2C9*3 is the most common reduced-function allele in Asian and Thai populations [44], no significant association with NSAID-induced SJS/TEN was observed in this study. This lack of association may be explained by the low allele frequency, limited statistical power, and the possibility that immune-mediated mechanisms play a more dominant role than metabolic pathways in the pathogenesis of NSAID-induced SJS/TEN. Other common variants, such as CYP2C9*2, CYP2C9*8, or other novel alleles, were not analyzed in this study because of their extremely low frequency (<1%) or absence in the Thai population [44], which would limit the statistical validity of making a comparison.

Although this study provides novel insights into the genetic variants associated with NSAID-induced SJS/TEN, particularly those related to piroxicam, several limitations should be acknowledged. First, the number of SJS/TEN cases was relatively small, reflecting the rarity of NSAID-induced SCARs and inherently limiting the statistical power to detect weaker associations. Second, the absence of an independent replication cohort restricts the generalizability and external validation of our findings. Third, only CYP2C9*3 was analyzed. Given that the CPIC guidelines for NSAID therapy based on the *CYP2C9* genotype incorporate multiple *CYP2C9* alleles, including decreased function alleles (CYP2C9*2, *5, *8, and *11) and no function alleles (CYP2C9*3, *6, and *13) [26], this narrow focus may underestimate the contribution of *CYP2C9* variability in NSAID-induced SJS/TEN. These limitations warrant cautious interpretation and highlight the need for replication in subsequent studies.

## 5. Conclusions

In conclusion, this study provides valuable preliminary evidence regarding the clinical characteristics and candidate pharmacogenetic factors associated with NSAID-induced SJS/TEN in the Thai population. HLA-DQB1*03:02 demonstrated the strongest suggestive association with NSAID-induced SJS/TEN, particularly piroxicam-related cases, representing an exploratory signal of a potential susceptibility marker. Additional alleles, including HLA-B*56:01, HLA-A*68:01, and HLA-DRB1*04:03, also showed suggestive associations; however, these findings should be interpreted with caution given the limited sample size and the loss of statistical significance after Bonferroni’s correction. No significant association was observed for CYP2C9*3, despite its relevance to NSAID metabolism. Notably, the strength of the association identified in this study appears to be weaker than the well-established *HLA* risk alleles reported for carbamazepine- or allopurinol-induced SJS/TEN, suggesting that additional genetic, immunologic, or environmental factors, such as T-cell receptor repertoire diversity, may influence susceptibility to NSAID-induced SJS/TEN. Given the rarity of SJS/TEN and the modest effect sizes observed, these findings lay important groundwork for future pharmacogenetic screening; however, broader validation is required before routine clinical implementation can be recommended. *HLA* testing may currently be most valuable in research settings or in carefully selected high-risk scenarios; therefore, validation in larger, multicenter, and ethnically diverse cohorts is essential to confirm these associations and establish their clinical utility.

## Figures and Tables

**Table 1:** Demographic, medical, and clinical data of patients with NSAID-induced SJS/TEN and tolerant controls enrolled in this study.

Characteristics	SJS	SJS/TEN Overlap	TEN	Total SJS/TEN	Tolerant Controls
Demographic Data					
*n*	14	1	3	18	54
Age, years					
Mean (SD)	52.21 (17.19)	70 (NA)	42.67 (25.70)	51.61 (18.38)	60.43 (10.12)
Median [range]	52 [31–95]	70	39 [19–70]	52 [19–95]	63 [38–82]
Sex, *n* (%)					
Male	6 (42.86)	1 (100)	2 (66.67)	9 (50)	10 (18.52)
Female	8 (57.14)	0 (0)	1 (33.33)	9 (50)	44 (81.48)
Medical data					
Onset of NSAID-induced SCARs, days					
Mean (SD)	8 (10.54)	1	5 (3.46)	7.06 (9.41)	>180
Median [range]	2 [1–30]	1	7 [1–7]	2 [1–30]	-
Internal organ involvement					
Kidney function					
*n* (available data)	9	1	3	13	
Renal insufficiency *	0 (0)	1 (100)	0 (0)	1 (7.69)	-
Liver function					
*n* (available data)	8	1	3	12	
AST (unit/L), *n* (%)					
normal level (12–32)	5 (62.50)	1 (100)	3 (100)	9 (75)	-
2- to 3-fold	2 (25)	0 (0)	0 (0)	2 (16.67)	-
>3-fold	1 (12.50)	0 (0)	0 (0)	1 (8.33)	-
ALT (unit/L), *n* (%)					
normal level (4–36)	5 (62.50)	0 (0)	1 (33.33)	6 (50)	-
2- to 3-fold	1 (12.50)	1 (100)	2 (66.67)	4 (33.33)	-
>3-fold	2 (25)	0 (0)	0 (0)	2 (16.67)	-
Hematologic abnormalities					
Eosinophils, *n* (%)					
*n* (available data)	10	1	3	14	
<5%	8 (80)	1 (100)	3 (100)	12 (85.71)	-
≥5%	2 (20)	0 (0)	0 (0)	2 (14.29)	-
Mucosal involvement, *n* (%)					
*n* (available data)	14	1	3	18	
Ocular	8 (57.14)	1 (100)	1 (33.33)	11 (61.11)	-
Oral	13 (92.86)	1 (100)	2 (66.67)	15 (83.33)	-
Genital	4 (28.57)	1 (100)	0 (0)	5 (27.78)	-
Outcome from SCARs					
Duration of hospital stay for the treatment of SCARs, days					
*n* (available data)	9	1	2	12	
Mean (SD)	14.22 (24.05)	86	7 (1.41)	19.00 (29.56)	-
Median [range]	6 [3–78]	86	7 [6–8]	6.5 [3–86]	-
Decreased cases, *n* (%)	0 (0)	0 (0)	0 (0)	0 (0)	-
Cost of treatment, THB ^#^					
*n* (available data)	8	1	2	11	
Mean (SD)	10,355.50 (10,797.40)	449,342	9844 (3719.38)	40,852.79 (117,854.09)	-
Median [range]	6650.50 [223–28,900]	449,342	9844 [7214–12,474]	7940.50 [223–449,342]	-

SJS, Stevens–Johnson syndrome; TEN, toxic epidermal necrolysis; AST, aspartate aminotransferase; ALT, alanine transaminase; THB, Thai baht. * The serum creatinine value was higher than 2.25 mg/dL when SCARs occurred; ^#^ cost of treatment during hospital stay.

**Table 2:** Clinical characteristics of patients with NSAID-induced SJS/TEN.

Sex	Age * (Years)	Phenotypes	Causative Drugs	Indication for NSAIDs	Co-Medication	Co-Morbidity
F	36	SJS	Piroxicam	Musculoskeletal pain	Dicloxacillin, chlorpheniramine	None
F	31	SJS	Ibuprofen	Fever	Ciprofloxacin	None
M	42	SJS	Piroxicam	Musculoskeletal pain	None	None
M	36	SJS	Diclofenac	Musculoskeletal pain	Indomethacin	None
F	52	SJS	Celecoxib	Osteoarthritis	Prednisolone, fluoxetine, lorazepam, tolperisone	Depression
F	95	SJS	Piroxicam	Musculoskeletal pain	Metformin	Diabetes mellitus
M	70	TEN	Metamizole	Musculoskeletal pain	NA	Lung cancer
F	19	TEN	Ibuprofen	Fever	Ciprofloxacin	None
M	39	TEN	Piroxicam	Musculoskeletal pain	None	None
F	59	SJS	Naproxen	Musculoskeletal pain	Gabapentin	None
F	68	SJS	Piroxicam	Musculoskeletal pain	Vitamin D supplement	Osteopenia
M	70	SJS/TEN	Piroxicam	Musculoskeletal pain	Mefenamic acid	NA
M	33	SJS	Piroxicam	Fever	None	None
M	48	SJS	Piroxicam	Osteoarthritis	None	None
M	56	SJS	Piroxicam	Osteoarthritis	None	None
F	56	SJS	Celecoxib	Musculoskeletal pain	Pregabalin, tramadol	None
M	52	SJS	Piroxicam	Musculoskeletal pain	NA	Cerebrovascular accident (old CVA)
F	67	SJS	Piroxicam	Osteoarthritis	Tolperisone	None

F, female; M, male; NA, not available; SJS, Stevens–Johnson syndrome; TEN, toxic epidermal necrolysis. * Age at the development of NSAID-induced SJS/TEN.

**Table 3:** List of odds ratios (ORs) and 95% confidence intervals (CIs) for NSAID-induced SJS/TEN in individuals carrying candidate *HLA* alleles compared with tolerant controls and the general Thai population.

*HLA* Alleles	NSAID- Induced SJS/TEN Cases (*n* = 18)	Tolerant Controls (*n* = 54)	General Thai Population ^a^ (*n* = 183)	NSAID-Induced SJS/TEN vs. Tolerant Controls			NSAID-Induced SJS/TEN vs. General Thai Population		
	Carriers (%)	Carriers (%)	Carriers (%)	OR [95% CI]	*p*-Value	Pc-Value ^#^	OR [95% CI]	*p*-Value	Pc-Value ^#^
*HLA* Class I									
A*68:01	3 (16.67)	2 (3.70)	4 (2.04)	5.20 [0.79–34.05]	0.0959	2.0139	**8.95** **[1.83–43.75]**	**0.0068**	0.1428
B*56:01	2 (11.11)	1 (1.85)	2 (1.10)	6.63 [0.56–77.91]	0.1522	5.3270	**11.31** **[1.49–85.75]**	**0.0189**	0.6615
*HLA* Class II									
DQB1*03:02	5 (27.78)	8 (14.81)	4 (4.00) ^b^	2.21 [0.62–7.92]	0.2886	3.7528	**9.23** **[2.19–38.83]**	**0.0024**	**0.0312**

*p*-values were calculated using Fisher’s exact test. All *p*-values and Pc-values < 0.05 are highlighted in bold. ^#^ The corrected *p*-values (Pc) for the multiple comparisons of all alleles were calculated using Bonferroni’s correction (21 for *HLA-A*, 35 for *HLA-B*, 19 for *HLA-C*, 22 for *HLA-DRB1*, 9 for *HLA-DQA1*, and 13 for *HLA-DQB1*). ^a^ Data obtained from published information [29]. ^b^ Data obtained from published information [32].

**Table 4:** List of odds ratios (ORs) and 95% confidence intervals (CIs) for piroxicam-induced SJS/TEN in individuals carrying candidate *HLA* alleles compared with tolerant controls and the general Thai population.

*HLA* Alleles	Piroxicam-Induced SJS/TEN Cases (*n* = 11)	Tolerant Controls (*n* = 54)	General Thai Population ^a^ (*n* = 183)	Piroxicam-Induced SJS/TEN vs. Tolerant Controls			Piroxicam-Induced SJS/TEN vs. General Thai Population		
	Carriers (%)	Carriers (%)	Carriers (%)	OR [95% CI]	*p*-Value	Pc-Value ^#^	OR [95% CI]	*p*-Value	Pc-Value ^#^
*HLA* Class I									
A*68:01	2 (18.18)	2 (3.70)	4 (2.04)	5.78 [0.72–46.42]	0.0990	2.0790	**9.94** **[1.60–61.66]**	**0.0136**	0.2856
B*56:01	2 (18.18)	1 (1.85)	2 (1.10)	11.78 [0.97–143.81]	0.0534	1.8690	**20.11** **[2.53–159.56]**	**0.0045**	0.1575
*HLA* Class II									
DRB1*04:03	2 (18.18)	0 (0)	3 (1.68)	**16.50** **[1.56–174.99]**	**0.0194**	0.4268	**13.33** **[1.97–90.07]**	**0.0079**	0.1738
DQB1*03:02	4 (36.36)	8 (14.81)	4 (4.00) ^b^	3.29 [0.78–13.86]	0.1054	1.3702	**13.71** **[2.81–66.86]**	**0.0012**	**0.0156**

*p*-values were calculated using Fisher’s exact test. All *p*-values and Pc-values < 0.05 are highlighted in bold. ^#^ The corrected *p*-values (Pc) for the multiple comparisons of all alleles were calculated using Bonferroni’s correction (21 for *HLA-A*, 35 for *HLA-B*, 19 for *HLA-C*, 22 for *HLA-DRB1*, 9 for *HLA-DQA1*, and 13 for *HLA-DQB1*). ^a^ Data obtained from published information [29]. ^b^ Data obtained from published information [32].

**Table 5:** List of odds ratios (ORs) and 95% confidence intervals (CIs) for NSAID-induced SJS/TEN, including subgroup analysis for piroxicam-induced SJS/TEN, in individuals carrying different CYP2C9*3 genotypes compared with tolerant controls and the general Thai population.

**Compared with the Tolerant Controls**							
CYP2C9*3 **Genotypes**	**Tolerant** **Controls** **(*n* = 54)**	**NSAID-Induced SJS/TEN** **(*n* = 18)**			**Piroxicam-Induced SJS/TEN** **(*n* = 11)**		
	**Carrier (%)**	**Carrier (%)**	**OR** **[95% CI]**	***p*** **-Value**	**Carrier (%)**	**OR** **[95% CI]**	***p*** **-Value**
CYP2C9*1/*1	51 (94.44)	17 (94.44)	Reference		10 (90.91)	Reference	
CYP2C9*1/*3	3 (5.56)	1 (5.56)	1.00 [0.10–10.27]	1.0000	1 (9.09)	1.70 [0.16–18.05]	0.6598
CYP2C9*3/*3	0 (0)	0 (0)	NA	NA	0 (0)	NA	NA
**Compared with the General Thai Population**							
CYP2C9*3 **Genotypes**	**General Thai Population** **(*n* = 183)**	**NSAID-Induced SJS/TEN** **(*n* = 18)**			**Piroxicam-Induced SJS/TEN** **(*n* = 11)**		
	**Carrier (%)**	**Carrier (%)**	**OR** **[95% CI]**	***p*** **-Value**	**Carrier (%)**	**OR** **[95% CI]**	***p*** **-Value**
CYP2C9*1/*1	168 (91.80)	17 (94.44)	Reference		10 (90.91)	Reference	
CYP2C9*1/*3	15 (8.20)	1 (5.56)	0.66 [0.08–5.30]	0.6948	1 (9.09)	1.12 [0.13–9.35]	0.9166
CYP2C9*3/*3	0 (0)	0 (0)	NA	NA	0 (0)	NA	NA

NA, not available.

## Data Availability

The original contributions presented in this study are included in the article/Supplementary Materials. Further inquiries can be directed to the corresponding author.

## Supplementary Materials

The following supporting information can be downloaded at https://www.mdpi.com/article/10.3390/medsci14010098/s1. Table S1: *HLA* class I allele frequencies (%AF) and number of carriers (%) of each allele observed in NSAID-induced SJS/TEN cases and tolerant controls; Table S2: *HLA* class II allele frequencies (%AF) and number of carriers (%) of each allele observed in NSAID-induced SJS/TEN cases and tolerant controls.

## Abbreviations

The following abbreviations are used in this manuscript:

ADRs	Adverse drug reactions
CYP2C9	Cytochrome P450 2C9
HLA	Human leukocyte antigen
MPE	Maculopapular exanthema
NSAIDs	Non-steroidal anti-inflammatory drugs
SCARs	Severe cutaneous adverse reactions
SJS	Stevens–Johnson syndrome
SOCs	Severe ocular surface complications
TEN	Toxic epidermal necrolysis
