# Supplementary material for: Genetic Variants Associated with Non-Steroidal Anti-Inflammatory Drug-Induced Stevens–Johnson Syndrome and Toxic Epidermal Necrolysis
Source: Med Sci (Basel). 2026 Feb 19;14(1):98. doi: 10.3390/medsci14010098 (PMC13028346; doi:10.3390/medsci14010098)
Supplement: Supplementary file 1 [file medsci-14-00098-s001.zip › medsci-4128335-supplementary.pdf]

Supplementary Tables

Table S1. *HLA* class I allele frequencies (%AF) and number of carriers (%) of each allele observed in NSAID-induced SJS/TEN cases and tolerant controls.

HLA class I alleles	SJS/TEN (n=18)		Tolerant controls (n=54)	
	AF (%)	Carrier (%)	AF (%)	Carrier (%)
HLA-A				
*A*11:01*	30.56	55.56	25.00	42.59
*A*02:01*	11.11	22.22	4.63	9.26
*A*02:03*	11.11	22.22	16.67	29.63
*A*02:07*	8.33	16.67	5.56	11.11
*A*68:01*	8.33	16.67	1.85	3.70
*A*24:02*	8.33	16.67	6.48	12.96
*A*24:07*	5.56	11.11	3.70	7.41
*A*24:10*	5.56	5.56	2.78	5.56
*A*33:03*	5.56	11.11	9.26	16.67
*A*02:06*	2.78	5.56	1.85	3.70
*A*33:01*	2.78	5.56	0.93	1.85
HLA-B				
*B*15:02*	11.11	22.22	8.33	12.96
*B*40:01*	11.11	16.67	5.56	11.11
*B*46:01*	8.33	16.67	14.81	25.93
*B*51:01*	8.33	16.67	2.78	5.56
*B*13:01*	5.56	11.11	5.56	9.26
*B*18:02*	5.56	11.11	2.78	5.56
*B*38:02*	5.56	11.11	2.78	5.56
*B*56:01*	5.56	11.11	0.93	1.85
*B*27:06*	2.78	5.56	0.93	1.85
*B*07:02*	2.78	5.56	1.85	3.70
*B*07:05*	2.78	5.56	9.26	18.52
*B*08:01*	2.78	5.56	1.85	3.70
*B*39:01*	2.78	5.56	0	0
*B*39:09*	2.78	5.56	4.63	9.26
*B*40:02*	2.78	5.56	1.85	3.70
*B*40:04*	2.78	5.56	0	0
*B*40:06*	2.78	5.56	0.93	1.85
*B*44:03*	2.78	5.56	0.93	1.85

HLA class I alleles	SJS/TEN (n=18)		Tolerant controls (n=54)	
	AF (%)	Carrier (%)	AF (%)	Carrier (%)
*B*48:01*	2.78	5.56	1.85	3.70
*B*54:01*	2.78	5.56	1.85	3.70
*B*55:04*	2.78	5.56	0	0
*B*58:01*	2.78	5.56	6.48	12.96
HLA-C				
*C*07:02*	19.44	27.78	19.44	33.33
*C*01:02*	16.67	33.33	17.59	31.48
*C*03:04*	8.33	11.11	3.70	5.56
*C*08:01*	8.33	16.67	10.19	16.67
*C*06:02*	5.56	11.11	2.78	5.56
*C*07:04*	5.56	11.11	5.56	11.11
*C*12:02*	5.56	11.11	4.63	9.26
*C*12:03*	5.56	11.11	2.78	5.56
*C*14:02*	5.56	11.11	3.70	7.41
*C*03:02*	2.78	5.56	7.41	14.81
*C*03:03*	2.78	5.56	2.78	5.56
*C*04:06*	2.78	5.56	1.85	3.70
*C*07:01*	2.78	5.56	1.85	3.70
*C*08:03*	2.78	5.56	0.93	1.85
*C*15:02*	2.78	5.56	1.85	3.70
*C*15:07*	2.78	5.56	0	0

Notes: The number of carriers of each allele observed in >1% of this population is presented in this Table. AF, allele frequency; Carriers, individuals who carried that allele.

Table S2. *HLA* class II allele frequencies (%AF) and number of carriers (%) of each allele observed in NSAID-induced SJS/TEN cases and tolerant controls.

HLA class II	SJS/TEN (n=18)		Tolerant controls (n=54)	
	AF (%)	Carrier (%)	AF (%)	Carrier (%)
HLA-DRB1				
*DRB1*15:02*	19.44	27.78	12.04	18.52
*DRB1*12:02*	13.89	22.22	13.89	25.93
*DRB1*04:05*	11.11	22.22	24.07	48.15
*DRB1*07:01*	8.33	16.67	1.85	3.70
*DRB1*03:01*	5.56	11.11	0.93	1.85
*DRB1*04:03*	5.56	11.11	0	0
*DRB1*14:04*	5.56	11.11	0.93	1.85
*DRB1*08:03*	2.78	5.56	0	0
*DRB1*09:01*	2.78	5.56	10.19	20.37
*DRB1*11:01*	2.78	5.56	0.93	1.85
*DRB1*12:01*	2.78	5.56	0	0
*DRB1*14:01*	2.78	5.56	1.85	3.70
*DRB1*14:05*	2.78	5.56	0.93	1.85
*DRB1*15:01*	2.78	5.56	10.19	14.81
*DRB1*15:05*	2.78	5.56	0	0
*DRB1*16:02*	2.78	5.56	5.56	9.26
HLA-DQA1				
*DQA1*01:01*	30.56	38.89	33.33	46.30
*DQA1*03:02*	13.89	27.78	12.04	24.07
*DQA1*01:02*	11.11	16.67	26.85	40.74
*DQA1*03:01*	11.11	22.22	15.74	29.63
*DQA1*05:05*	8.33	16.67	1.85	3.70
*DQA1*06:01*	8.33	16.67	6.48	12.96
*DQA1*01:03*	5.56	11.11	0	0
*DQA1*02:01*	5.56	11.11	1.85	3.70
*DQA1*05:01*	5.56	11.11	1.85	3.70
HLA-DQB1				
*DQB1*03:01*	16.67	27.78	9.26	18.52
*DQB1*03:02*	13.89	27.78	8.33	14.81
*DQB1*05:01*	16.67	27.78	24.07	42.59
*DQB1*05:02*	11.11	22.22	14.81	25.93
*DQB1*06:01*	11.11	22.22	8.33	14.81
*DQB1*02:02*	8.33	16.67	1.85	3.70

HLA class II	SJS/TEN (n=18)		Tolerant controls (n=54)	
	AF (%)	Carrier (%)	AF (%)	Carrier (%)
*DQB1*04:01*	8.33	16.67	15.74	31.48
*DQB1*02:01*	5.56	11.11	0.93	1.85
*DQB1*05:03*	5.56	11.11	2.78	5.56
*DQB1*03:03*	2.78	5.56	11.11	22.22

Notes: The number of carriers of each allele observed in >1% of this population is presented in this Table. AF, allele frequency; Carriers, individuals who carried that allele.
